# Supplementary material for: Structural mechanism of FusB-mediated rescue from fusidic acid inhibition of protein synthesis
Source: Nat Commun. 2025 Apr 18;16:3693. doi: 10.1038/s41467-025-58902-3 (PMC12008383; doi:10.1038/s41467-025-58902-3)
Supplement: Supplementary file 1 — Supplementary Information [file 41467_2025_58902_MOESM1_ESM.pdf]

Supplementary material for:

Structural mechanism of FusB-mediated rescue from fusidic acid inhibition of protein synthesis

Adrián González-López[1,2], Xueliang Ge[1], Daniel S.D. Larsson[1], Carina Sihlbom Wallem[3], Suparna Sanyal[1], and Maria Selmer[1,2]*

1. Department of Cell and Molecular Biology, Uppsala University, BMC, P.O. Box 596, SE-75124 Uppsala, Sweden
2. Uppsala Antibiotic Center, Uppsala University, Sweden
3. Proteomics Core Facility, Scilifelab and University of Gothenburg, SE-405 30 Gothenburg, Sweden

*To whom correspondence should be addressed: maria.selmer@icm.uu.se,

tel. +46 18 4714177

Content:

Supplementary Figures 1-17

Supplementary Tables 1-2

Supplementary References

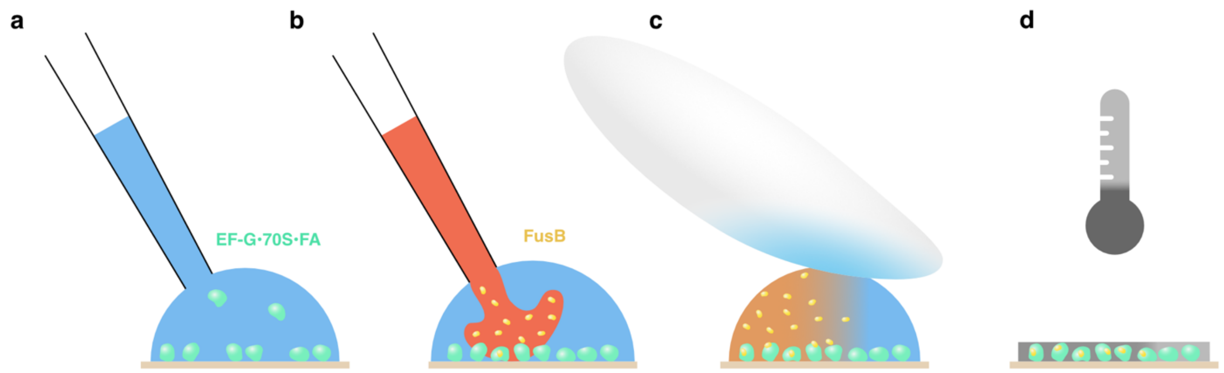

Supplementary Figure 1. Schematic of the mix-on-grid method. **(a)** The first component, EF-G•FA•70S (green) is pipetted onto the grid and incubated to allow binding to the continuous carbon. **(b)** The second component, FusB (orange), is pipetted into the drop on the grid. **(c)** Blotting is immediately performed in the Vitrobot Mark IV. **(d)** The sample is vitrified in liquid ethane.

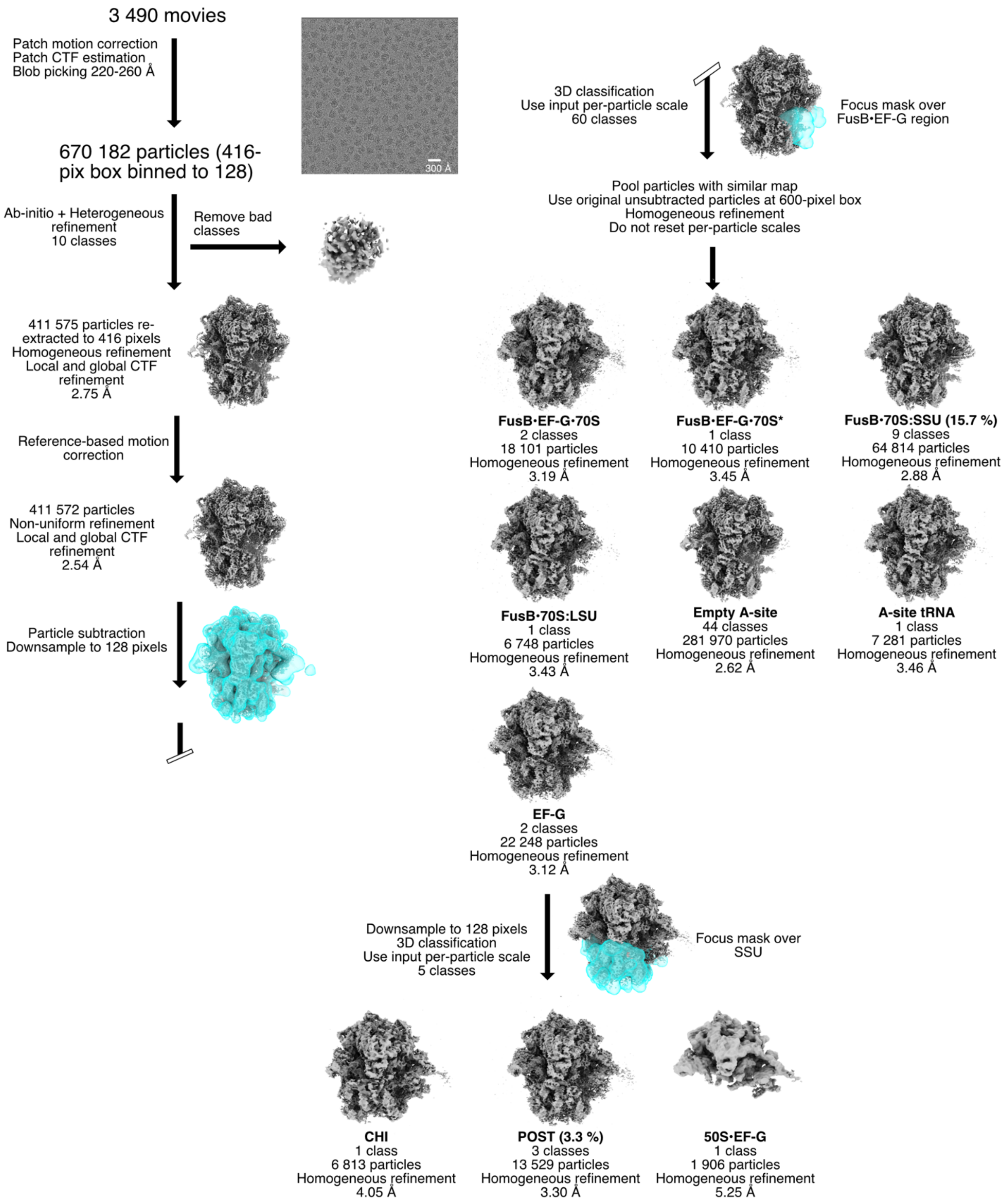

Supplementary Figure 2. Processing workflow for preliminary dataset1 using cryoSPARC v4.4.1. Percentage of total 70S particles in FusB•70S:SSU and POST are indicated.

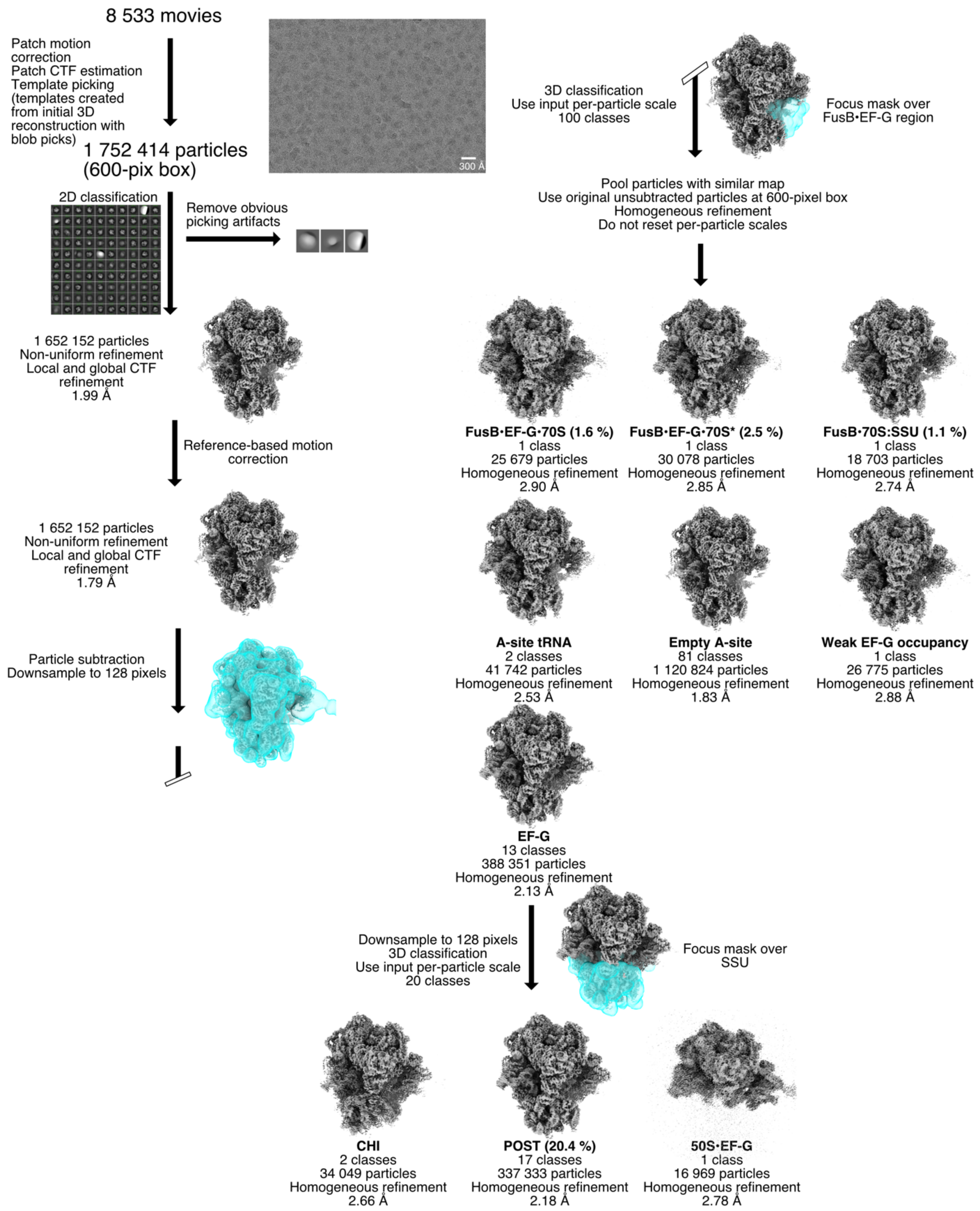

Supplementary Figure 3. Processing workflow for preliminary dataset2 using cryoSPARC v4.4.1. Percentage of total 70S particles in FusB•70S:SSU and POST are indicated.

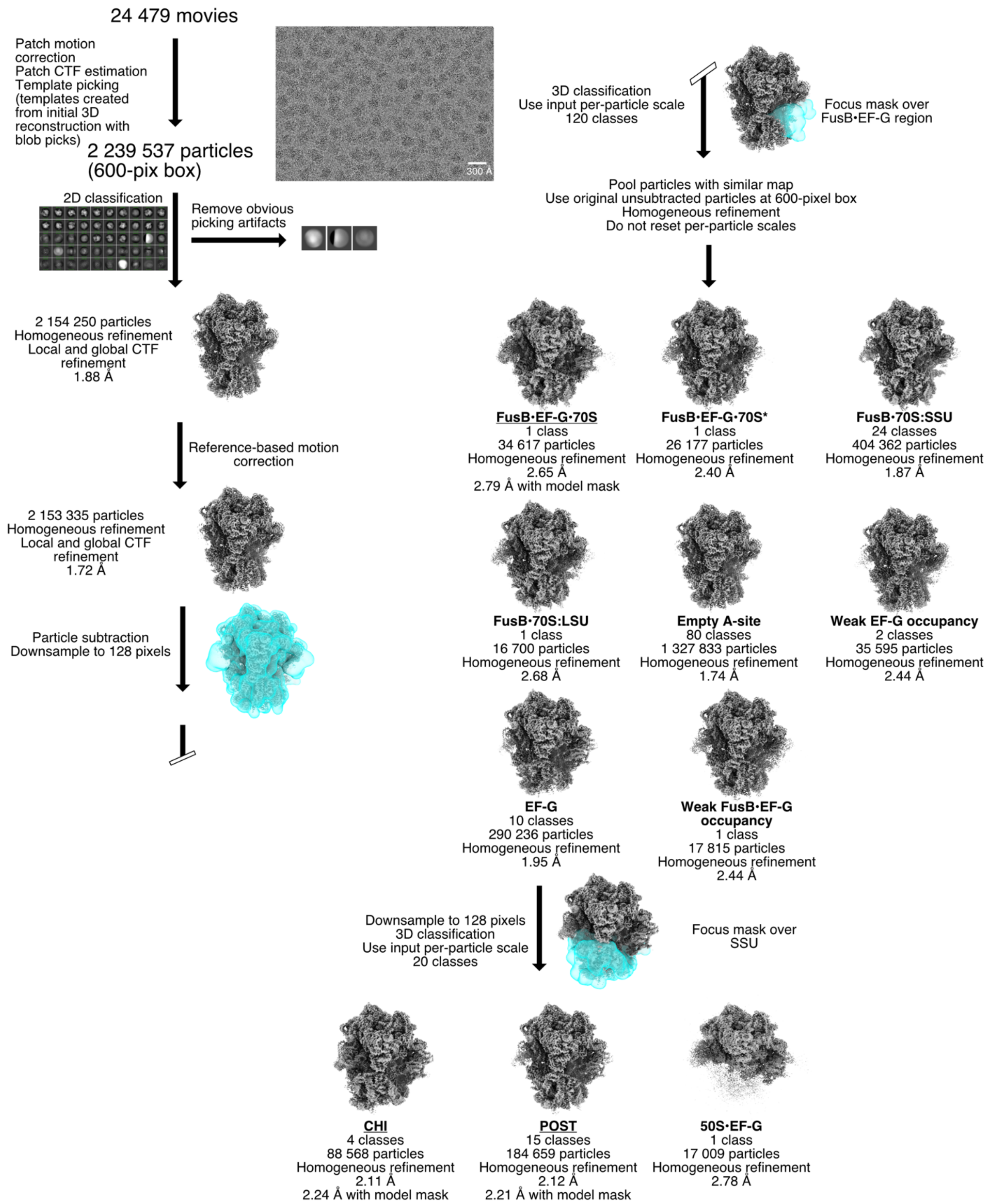

Supplementary Figure 4. Processing workflow for the early dataset (6 s) using cryoSPARC v4.4.1. Maps used for model building and refinement are underlined.

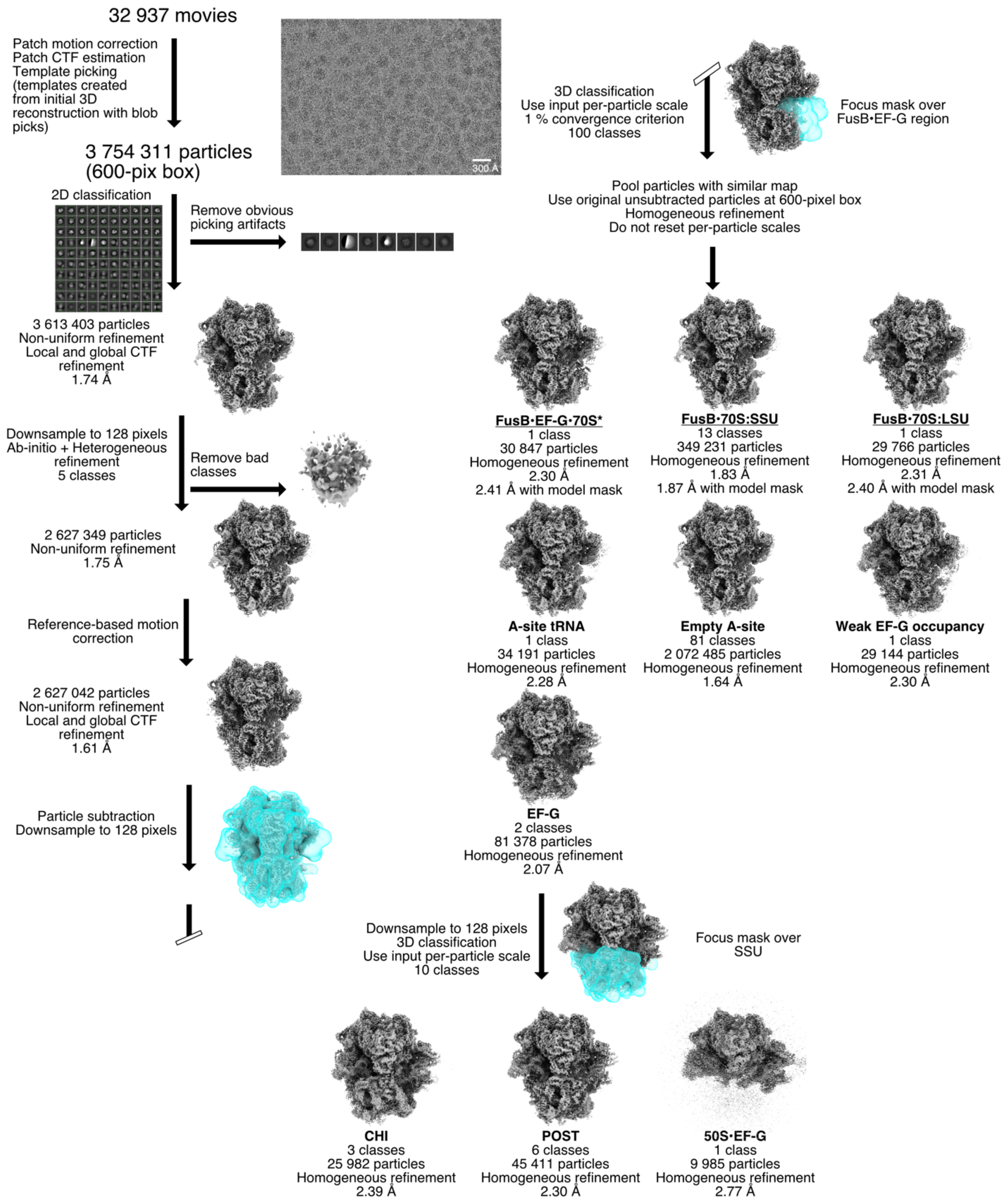

Supplementary Figure 5. Processing workflow for the 25 s dataset using cryoSPARC v4.4.1. Maps used for model building and refinement are underlined.

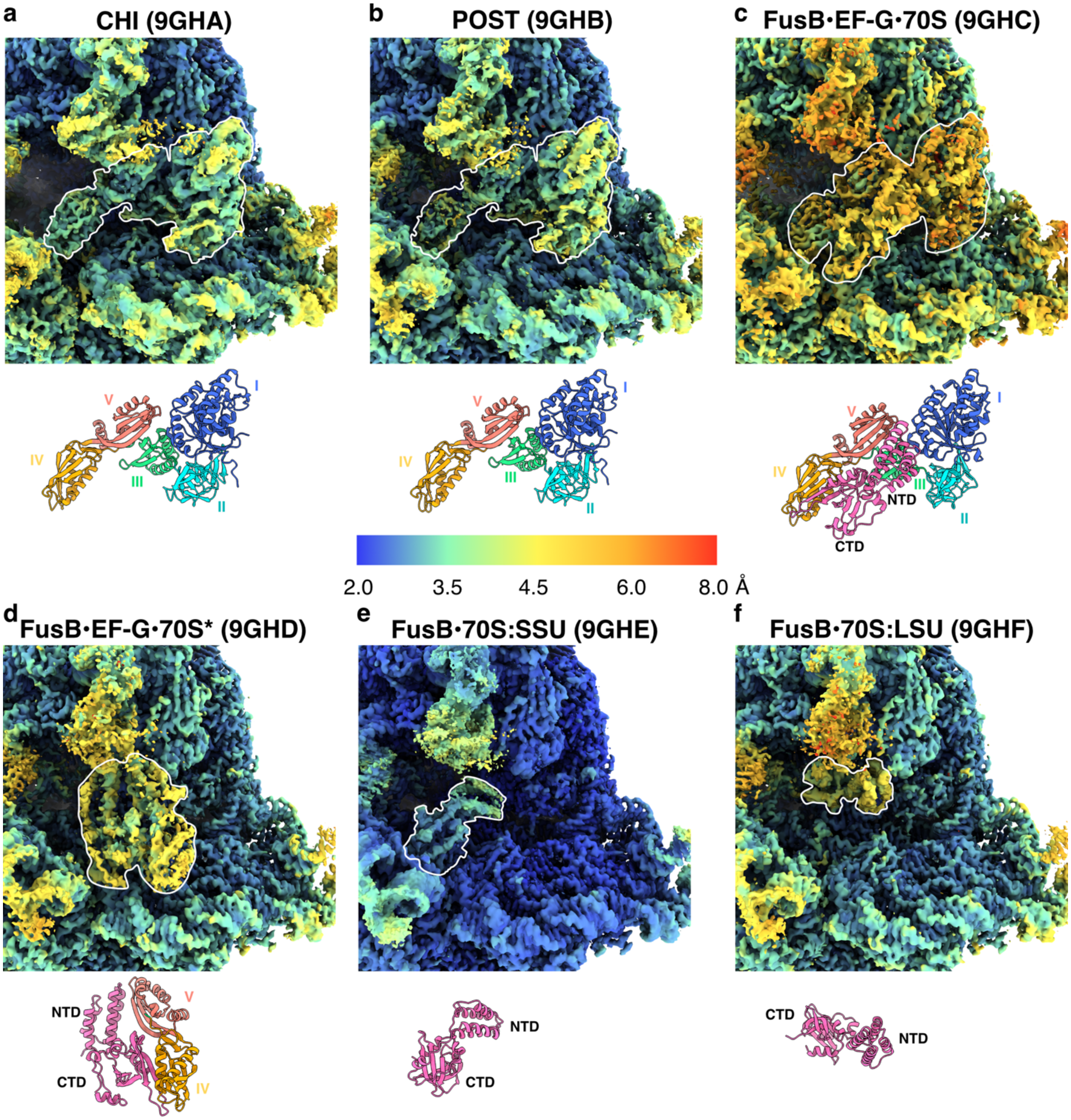

Supplementary Figure 6. Cryo-EM maps around the ribosomal A-site from the early and 25 s datasets colored by local resolution estimation from cryoSPARC. The cartoon representation of the EF-G (colored by domain) and FusB (pink) models in the same orientation is shown below each panel. FusB and EF-G are outlined on the maps. **(a)** CHI. **(b)** POST. **(c)** FusB•EF-G•70S. **(d)** FusB•EF-G•70S*. **(e)** FusB•70S:SSU. **(f)** FusB•70S:LSU.

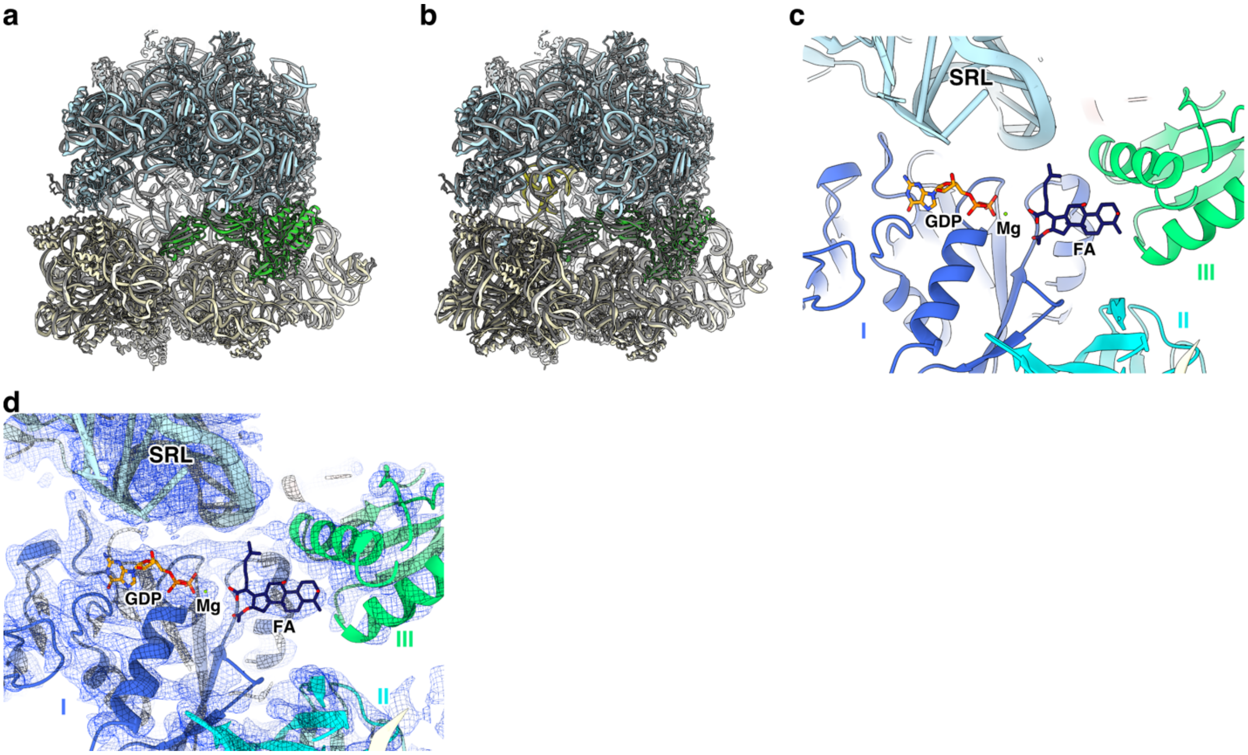

Supplementary Figure 7. (a) Comparison between the CHI state with *S. aureus* EF-G and *E. coli* 70S (LSU, light blue; SSU, light yellow; tRNA, yellow; EF-G, green), and *S. aureus* 70S (PDBID: 8P2H, gray)[1]. **(b)** Comparison between the POST state with *S. aureus* EF-G and *E. coli* 70S and *S. aureus* 70S (PDBID: 8P2F)[1]. Colors as in a. The structures are aligned by 23S rRNA. **(c)** FA-binding pocket in POST-state EF-G (colored by domain) with FA (dark blue), LSU (light blue), GDP (orange) and Mg (green). **(d)** Locally filtered map over panel c.

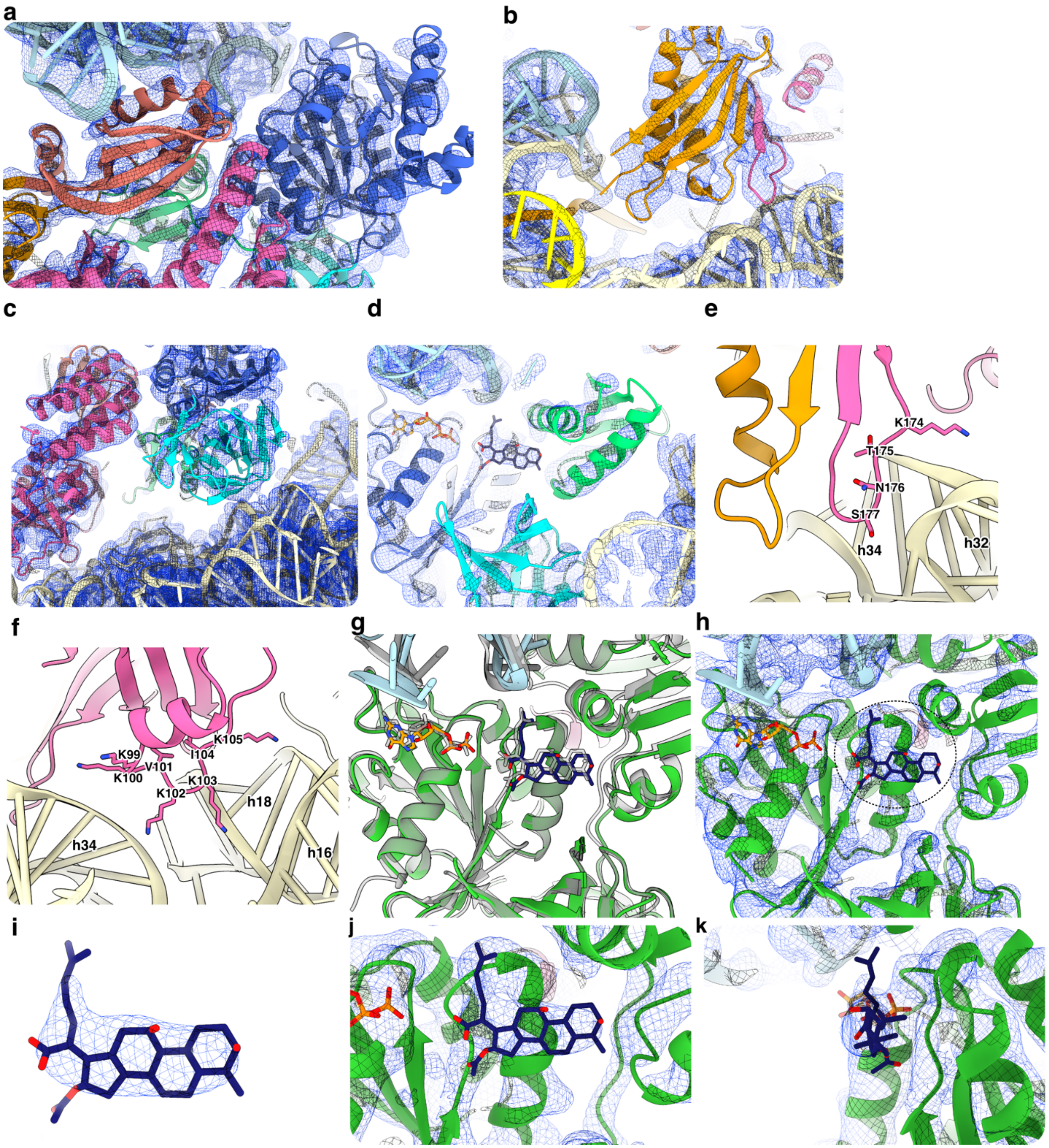

Supplementary Figure 8. Locally filtered map of FusB•EF-G•70S. **(a)** As Figure 2d. **(b)** As Figure 2e. **(c)** As Figure 2f. **(d)** As Figure 2g. **(e,f)** Loops of FusB interacting with the SSU (colors as in Figure 2). **(g)** Structure alignment of domains I-II of EF-G between POST (gray) and FusB•EF-G•70S showing the FA-binding pocket, with LSU (light blue), EF-G (green), FA (dark blue), and GDP (orange). **(h)** As panel g, with locally filtered map shown. **(i)** Segmented map of FusB•EF-G•70S 2 Å around FA. **(j)** As panel h, zoomed to FA. **(k)** As panel j, rotated 90°.

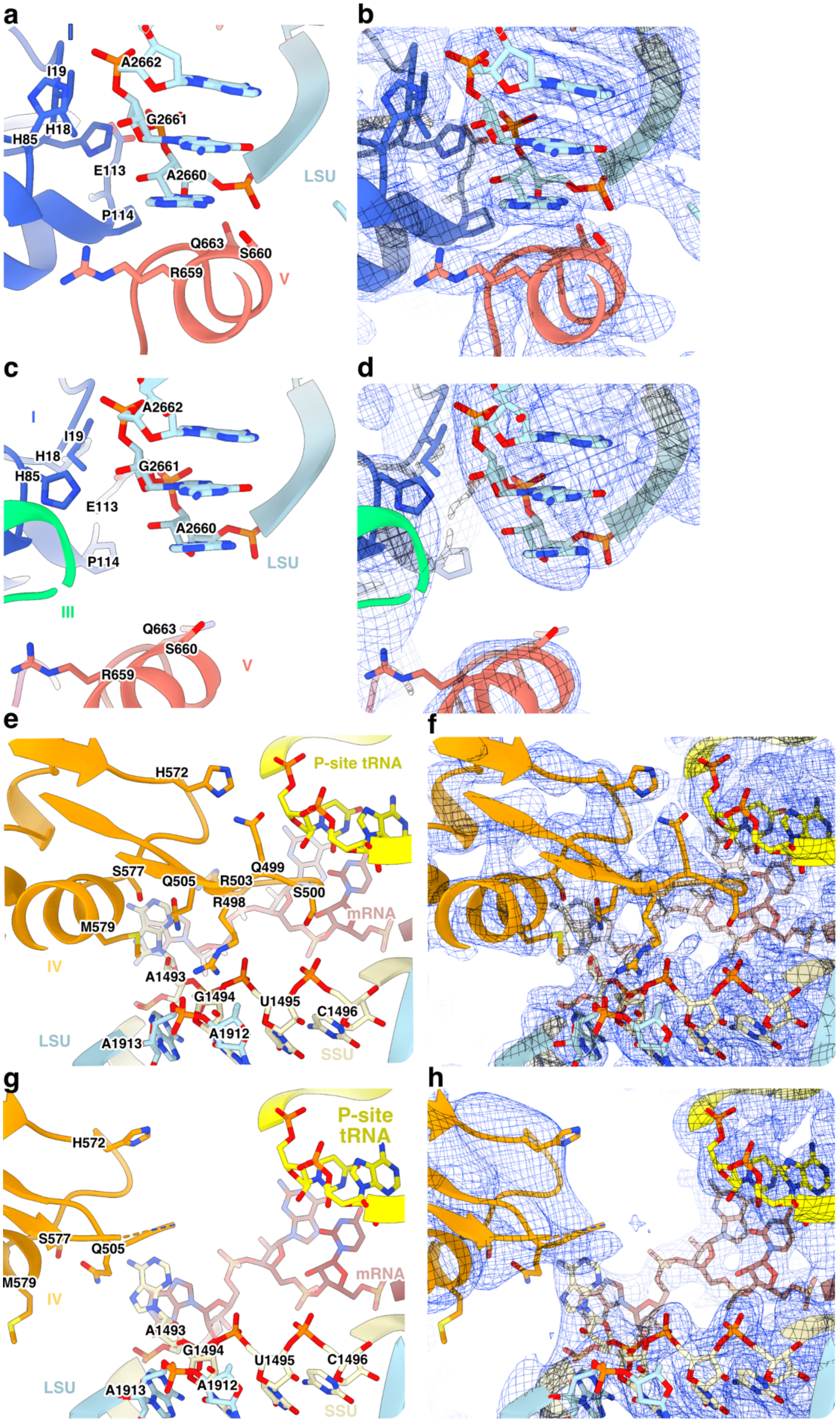
a
I
I19
A2662
H18
G2661
H85
E113
A2660
LSU
P114
Q663
S660
R659
V
b
c
A2662
I
I19
H18
G2661
H85
E113
A2660
P114
LSU
III
Q663
S660
R659
V
d
e
H572
P-site tRNA
S577
Q505
R503
Q499
S500
R498
mRNA
M579
IV
A1493
G1494
U1495
C1496
LSU
A1913
A1912
SSU
f
g
H572
P-site tRNA
S577
Q505
mRNA
M579
IV
A1493
G1494
U1495
C1496
LSU
A1913
A1912
SSU
h

Supplementary Figure 9. Binding of FusB leads to reduced contacts between EF-G and the ribosome, tRNA and mRNA (colors as in Figure 2) **(a-d)** Interaction of EF-G domain I and V with LSU. **(a)** POST structure **(b)** As panel a, showing locally filtered map. **(c)** FusB•EF-G•70S. **(d)** As panel c, showing locally filtered map. **(e-h)** Interaction of EF-G domain IV with the decoding center, P-site tRNA and mRNA. **(e)** POST structure **(f)** As panel e, showing locally filtered map. **(g)** FusB•EF-G•70S. **(h)** As panel g, showing locally filtered map.

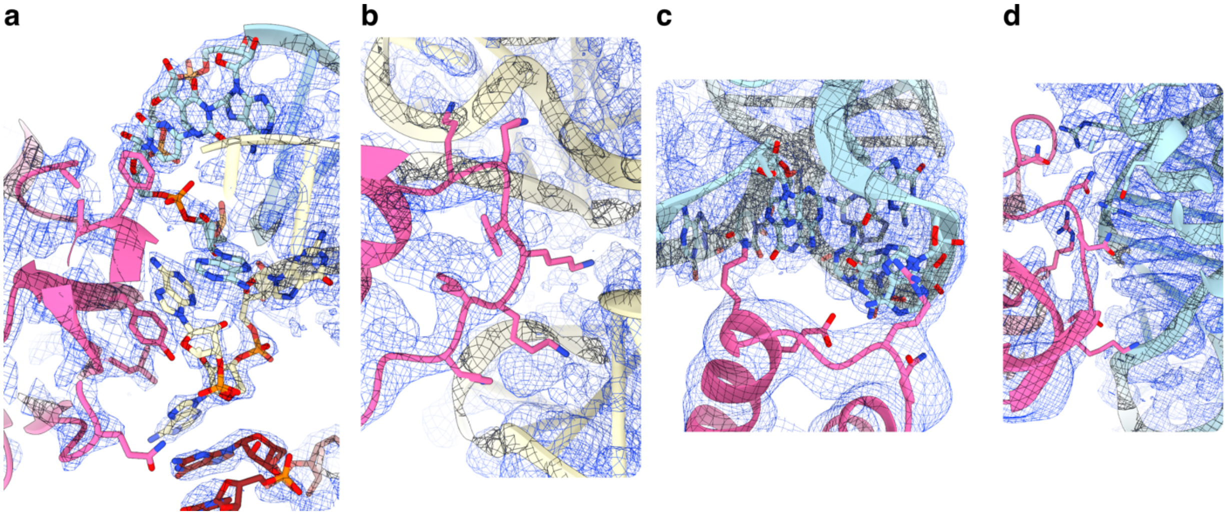

Supplementary Figure 10. Locally filtered maps of FusB•SSU and FusB•LSU around the regions shown in Figure 3b, c, e and f. **(a)** As Figure 3b. **(b)** As Figure 3c. **(c)** As Figure 3e. **(d)** As Figure 3f.

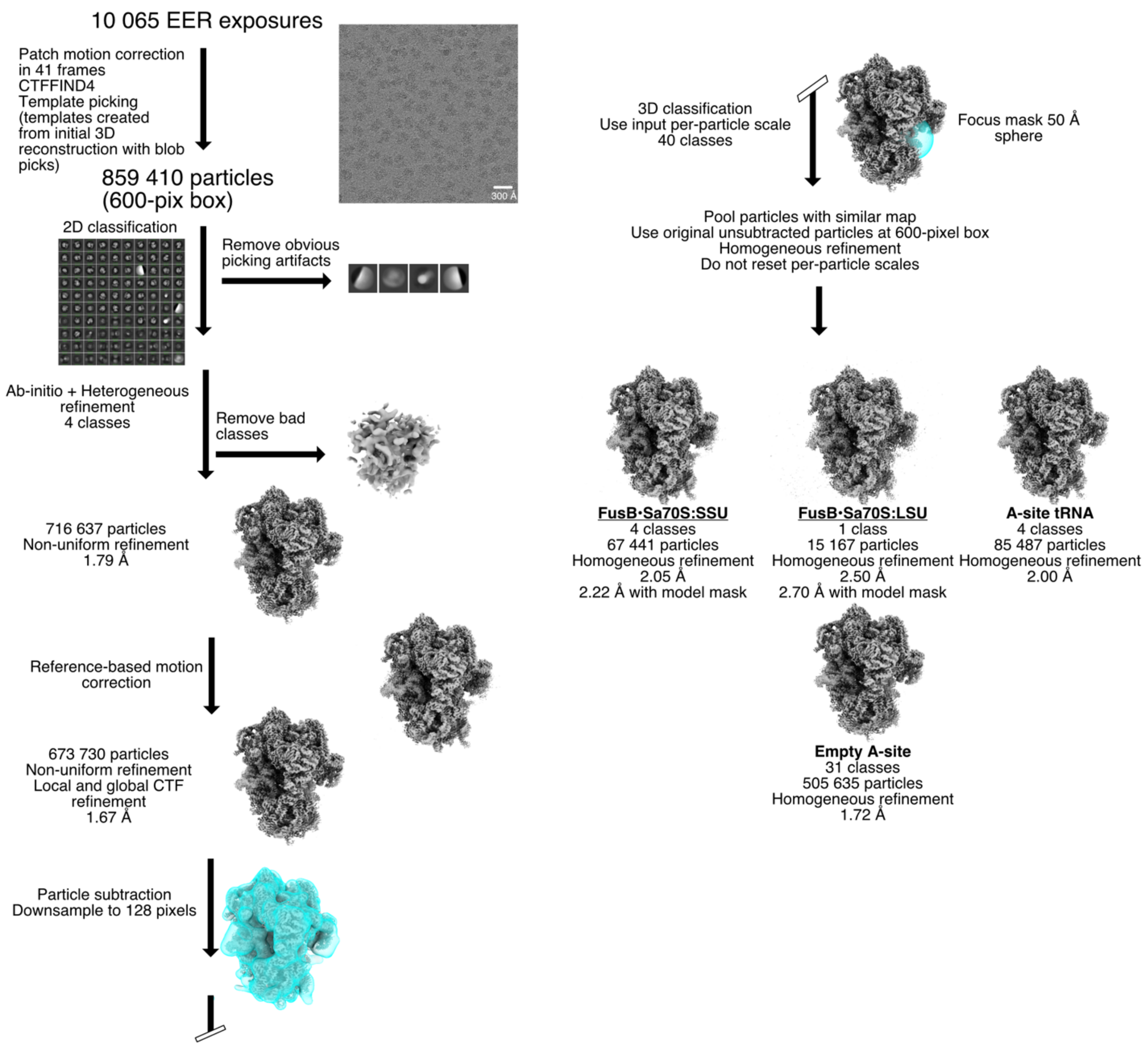

Supplementary Figure 11. Processing workflow for the FusB•*Sa*70S dataset using cryoSPARC v4.4.1. Maps used for model building and refinement are underlined.

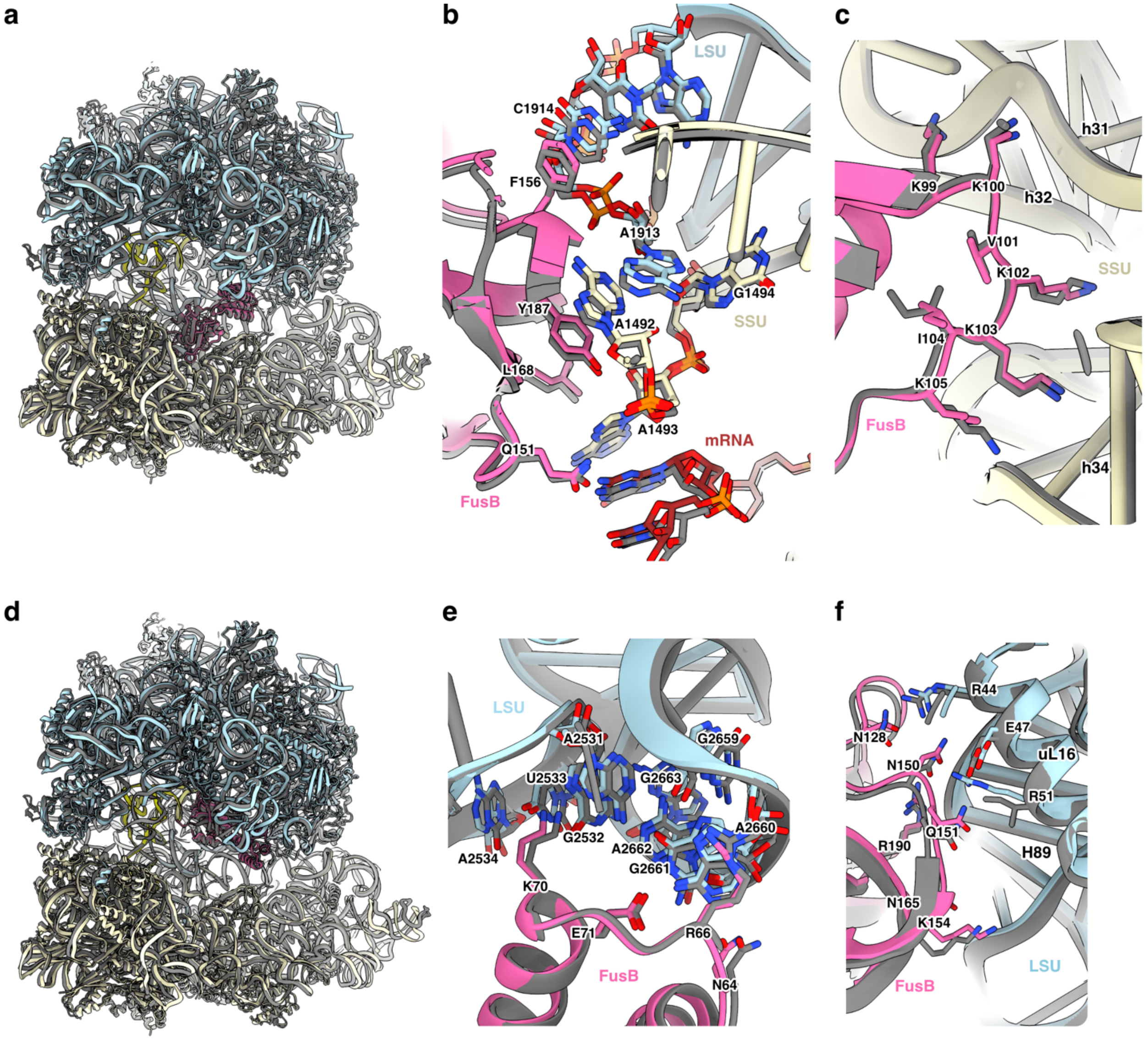

Supplementary Figure 12. Comparison of FusB complexes with *E. coli* (multicolor) and *S. aureus* (gray) 70S **(a)** Overlay of FusB•70S:SSU (LSU, light blue; SSU, light yellow; tRNA, yellow; FusB, pink), and FusB•Sa70S:SSU (gray). **(b)** Same interaction as in Figure 3b. **(c)** Same interaction as in Figure 3c. **(d)** Overlay of FusB•70S:LSU (colors as in panel a), and FusB•Sa70S:LSU (gray). **(e)** Same interaction as in Figure 3e. **(f)** Same interaction as in Figure 3f.

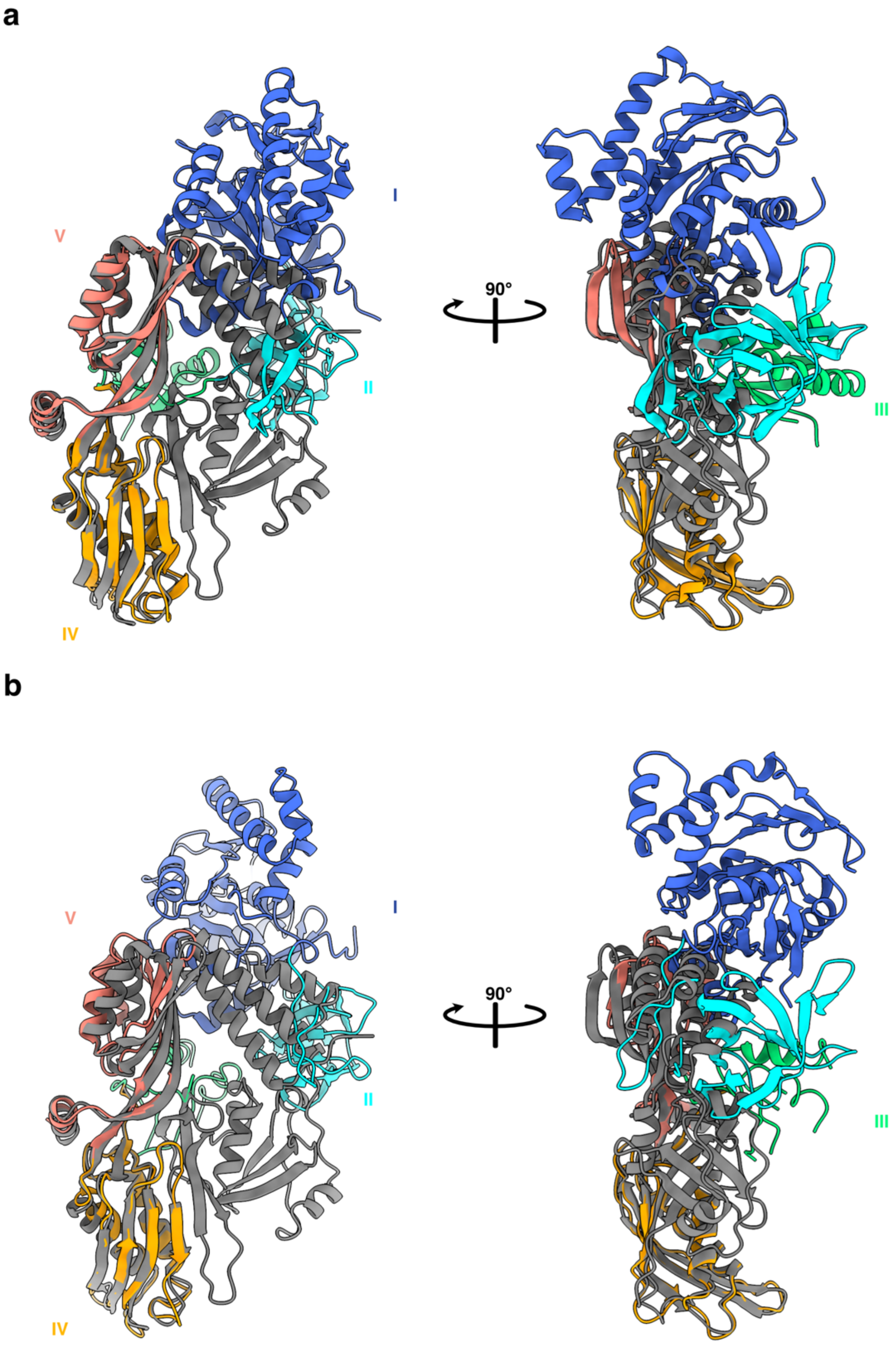

Supplementary Figure 13. Structure alignment based on domains IV-V of EF-G between FusB•EF-G in FusB•EF-G•70S (gray) with crystal structures of EF-G (colored by domain) shows that both domains of FusB would clash with domains I-II and the linker regions between domains II, III and IV. (**a**) Comparison between FusB•EF-G and the crystal structure of *S. aureus* EF-G (PDBID: 2XEX)[2]. **(b)** Comparison between FusB•EF-G and the crystal structure of *Thermus thermophilus* EF-G (PDBID: 1ELO)[3].

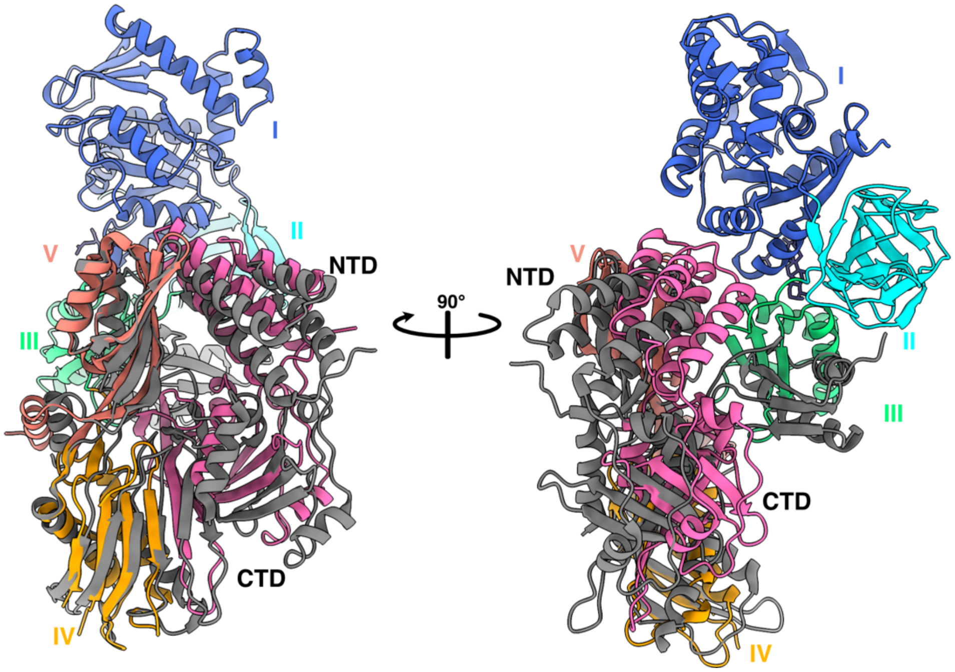

Supplementary Figure 14. Structure alignment based on domains IV-V of EF-G between the NMR model of FusB and EF-G domains I-III (gray, PDBID: 2MZW)[4] with FusB•EF-G in FusB•EF-G•70S (FusB, pink; EF-G, colored by domain). This results in an RMSD of 14.6 Å over 213 Cα atoms for FusB.

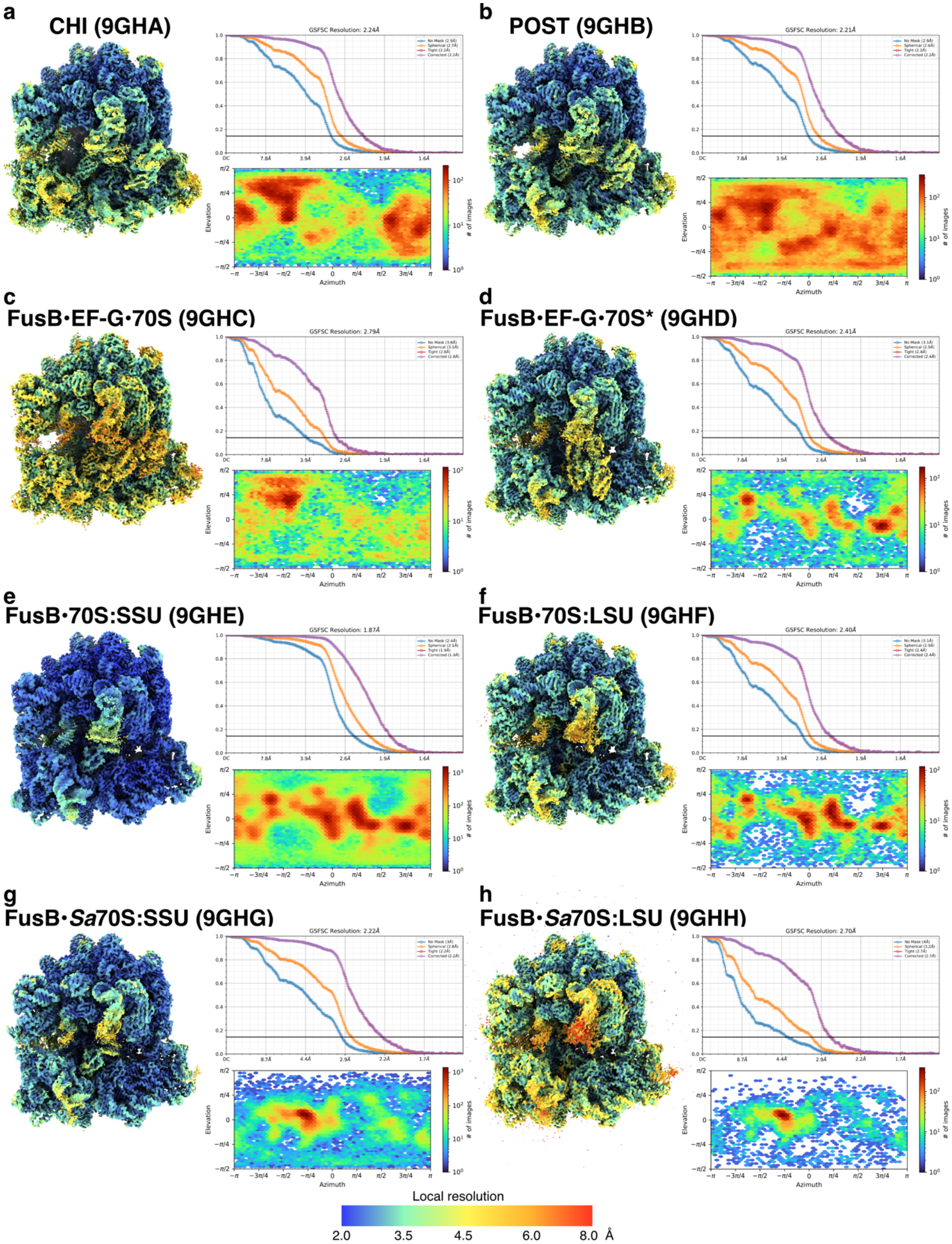

Supplementary Figure 15. (a-h) Local resolution and angular distribution of the particles for the maps used for modeling.

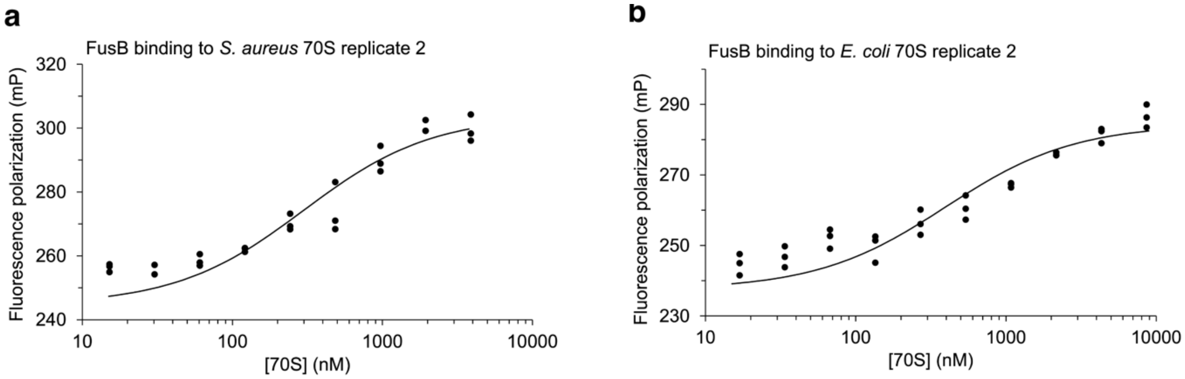

Supplementary Figure 16. Independent technical replicate of fluorescence polarization binding experiments measuring affinities of fluorescein-labeled FusB to 70S ribosomes from *S. aureus* **(a)** and *E. coli* **(b)** in n=3 independent measurements. Source data are provided as a Source Data file.

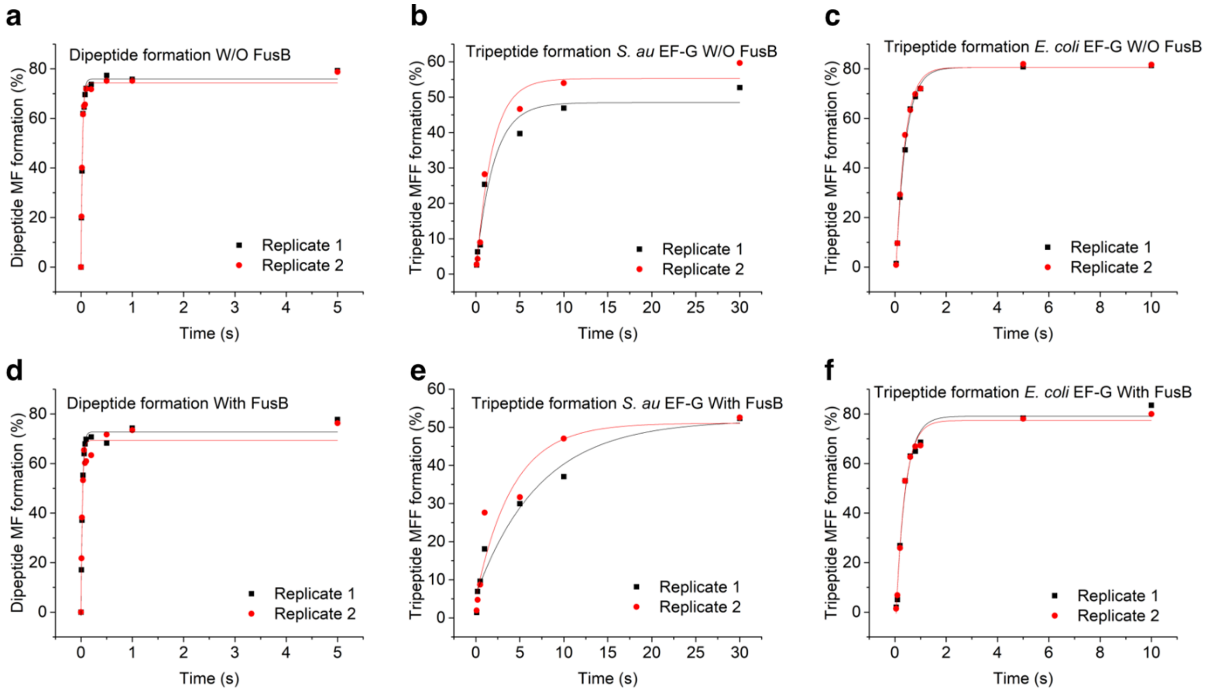

Supplementary Figure 17. Individual di- and tripeptide formation experiments in absence and presence of FusB (Figure 4), fitted to a single exponential curve. **(a-c)** without FusB, **(d-f)** with FusB. Source data are provided as a Source Data file.

Supplementary Table 1. Interface areas of EF-G in POST and FusB•EF-G•70S structures calculated by PDBePISA.

Chain 1	Chain 2	Interface area in **POST** (PDB 9GHB) (Å^2)	Interface area in **FusB•EF-G •70S** (PDB 9GHC) (Å^2)	Relative interface area in **FusB•EF-G •70S** compared to **POST**
W (EF-G)	V (FusB)	-	1959.4	
W (EF-G)	a (23S)	742.5	500.6	67%
W (EF-G)	A (16S)	526.4	305.5	58%
W (EF-G)	L (uS12)	176.6	161.5	91%
W (EF-G)	g (uL6)	126.3	113.2	90%
W (EF-G)	9 (mRNA)	149.7	0	0%
W (EF-G)	Z (P-site tRNA)	207.8	0	0%
Total		1929.3	3040.2	56%
Total ribosome		1571.8	1080.8	69%

Supplementary Table 2. Mass spectrometry results of total abundance for the relative quantification of FusB.

Pure recombinant FusB and EF-G with known concentrations were used for relative quantification in the *S. aureus* strain AH027. All proteins were digested with trypsin and peptides labeled with isobaric mass tagging using TMTpro (recombinant EF-G peptides 133C, recombinant FusB peptides 126, *S. aureus* lysate peptides 135N). The labelled peptides were pooled into different mixtures of TMTpro 3-plex sets based on the abundance intensity of previous run and the peptide standards compared to the *S. aureus* cell sample. Peptide standard concentrations are specified in the molecular ratio. Protein molecular ratio in the *S. aureus* lysate were calculated based on the added recombinant protein concentration and the summed detected peptide abundances. The reported mean value and standard deviation is based on all five individual mass spectrometry experiments (in nine runs), all from the same *S. aureus* pellet.

MS files	Uniprot code	Description	Coverage (%)	Peptides	PSMs	Unique Peptides	MW (kDa)	Sequest score	Abundances label 126, Spike Far1	Abundances label 133C, Spike EFG	Abundances label 135N, Cell lysate	Abundances label 126, Spike Far1	Abundances label 133C, Spike EFG	Abundances label 135N, Cell lysate	Average Far1 (FusB) Abundance	Average EFG Abundance	Average Cell Abundance	Mix aim Abundance STD:Cell	Protein concentration STD (pg/µl)	(STD conc)/(abun)*cell abun	Conc µmol/µl cell	Molar Ratio EFG:FusB
Eclipse_241001_31	Q2YSB4	Elongation factor G	34	15	45	15	76.6	196		1247	13784					1246.6	13784.3	1st test	0.164	$1.81*10^{-3}$	23.7	1.8
Eclipse_241001_31	Q8GNY5	Far1 (FusB)	41	9	24	9	25.2	74	21935		7118				21935		7117.8	1st test	0.9984	$3.24*10^{-4}$	12.9	
Lumos_241004_14, Lumos_241004_15	Q2YSB4	Elongation factor G	67	32	158	32	76.6	683		13768	13944		9508	10556		11638	12250	1:1	1.64	$1.73*10^{-3}$	22.5	2.4
Lumos_241004_14, Lumos_241004_15	Q8GNY5	Far1 (FusB)	55	11	19	11	25.2	67	4614		5429	2579		3156	3597		4293	1:1	0.19968	$2.38*10^{-4}$	9.46	
Lumos_241004_18, Lumos_241004_19	Q2YSB4	Elongation factor G	62	27	115	27	76.6	545		7882	15860		5761	12305		6822	14082	1:2	0.82	$1.69*10^{-3}$	22.1	2.4
Lumos_241004_18, Lumos_241004_19	Q8GNY5	Far1 (FusB)	51	10	19	10	25.2	66	2582		5481	1991		5263	2286		5372	1:2	0.09984	$2.35*10^{-4}$	9.31	
Lumos_241004_22, Lumos_241004_23	Q2YSB4	Elongation factor G	71	33	173	33	76.6	730		17528	12190		17483	11164		17506	11677	2:1	3.28	$2.19*10^{-3}$	28.6	2.3
Lumos_241004_22, Lumos_241004_23	Q8GNY5	Far1 (FusB)	55	11	28	11	25.2	94	5953		5277	7143		4952	6548		5114	2:1	0.39936	$3.12*10^{-4}$	12.4	
Lumos_241104_23, Lumos_241104_24	Q2YSB4	Elongation factor G	58	24	124	24	76.6	664		13043	17199		11110	14406		17506	13724	1:1	1.64	$1.29*10^{-3}$	16.8	2.7
Lumos_241104_23, Lumos_241104_24	Q8GNY5	Far1 (FusB)	38	8	18	8	25.2	62	4572		3775	5114		5189	6548		5114	1:1	0.19968	$1.56*10^{-4}$	6.19	

Supplementary References

1. González-López, A. *et al.* Structures of the Staphylococcus aureus ribosome inhibited by fusidic acid and fusidic acid cyclopentane. *Sci Rep* **14**, 14253 (2024).
2. Chen, Y., Koripella, R. K., Sanyal, S. & Selmer, M. *Staphylococcus aureus* elongation factor G – structure and analysis of a target for fusidic acid. *FEBS J* **277**, 3789–3803 (2010).
3. Ævarsson, A. *et al.* Three-dimensional structure of the ribosomal translocase: elongation factor G from *Thermus thermophilus*. *EMBO J* **13**, 3669–3677 (1994).
4. Tomlinson, J. H., Thompson, G. S., Kalverda, A. P., Zhuravleva, A. & O'Neill, A. J. A target-protection mechanism of antibiotic resistance at atomic resolution: insights into FusB-type fusidic acid resistance. *Sci Rep* **6**, 19524 (2016).
