# Supplementary material for: Structural mechanism of FusB-mediated rescue from fusidic acid inhibition of protein synthesis
Source: Nat Commun. 2025 Apr 18;16:3693. doi: 10.1038/s41467-025-58902-3 (PMC12008383; doi:10.1038/s41467-025-58902-3)
Supplement: Supplementary file 2 — Description of Additional Supplementary Files [file 41467_2025_58902_MOESM2_ESM.pdf]

Description of Additional Supplementary Files

File name: Supplementary Data 1
Description: Mass spectrometry results per detected peptide for the relative quantification of FusB. The recombinant proteins and the cell culture were analyzed in parallel (3-plex mix) in nine runs in total. The abundance of the labels from the MS3 spectra is reported per detected peptide per data-dependent acquisition (DDA) run, for the spiked protein or cell culture. Different peptides per protein is usually detected in each DDA run, but it is always the same peptide sequence in each sample, that is used per run for the total abundance reported in Supplementary Table 2.

File name: Supplementary Movie 1
Description: Morph showing the conformational changes of EF-G from the POST state to the FusB•EF-G•70S complex induced by binding of FusB, with structures aligned by 23S rRNA (colors as in Figure 2).
